# Genomic analysis of *Enterococcus faecium* strain RAOG174 associated with acute chorioamnionitis carried antibiotic resistance gene: is it time for precise microbiological identification for appropriate antibiotic use?

**DOI:** 10.1186/s12864-023-09511-1

**Published:** 2023-07-19

**Authors:** Pisut Pongchaikul, Roberto Romero, Paninee Mongkolsuk, Pornpun Vivithanaporn, Thidathip Wongsurawat, Piroon Jenjaroenpun, Perapon Nitayanon, Iyarit Thaipisuttikul, Threebhorn Kamlungkuea, Arunee Singsaneh, Pitak Santanirand, Piya Chaemsaithong

**Affiliations:** 1 Chakri Naruebodindra Medical Institute, Faculty of Medicine Ramathibodi Hospital Mahidol University, Samut Prakan, Thailand; 2 Integrative Computational BioScience Center, Mahidol University, Nakhon Pathom, Thailand; 3 Institute of Infection, Veterinary and Ecological Sciences, University of Liverpool, Liverpool, UK; 4 Pregnancy Research Branch (formerly The Perinatology Research Branch, NICHD/NIH/DHHS, in Detroit, Michigan, USA, has been renamed as the Pregnancy Research Branch, NICHD/NIH/DHHS), Division of Obstetrics and Maternal-Fetal Medicine, Division of Intramural Research, Eunice Kennedy Shriver National Institute of Child Health and Human Development, National Institutes of Health, United States Department of Health and Human Services, Bethesda, MD USA; 5 Division of Obstetrics and Maternal-Fetal Medicine, Division of Intramural Research, Eunice Kennedy Shriver National Institute of Child Health and Human Development, National Institutes of Health, United States Department of Health and Human Services, Detroit, MI USA; 6 Department of Obstetrics and Gynecology, University of Michigan, Ann Arbor, MI USA; 7 Department of Epidemiology and Biostatistics, Michigan State University, East Lansing, MI USA; 8 Division of Medical Bioinformatics, Research Department, Faculty of Medicine Siriraj Hospital, Mahidol University, Bangkok, Thailand; 9 Department of Microbiology, Faculty of Medicine Siriraj Hospital, Mahidol University, Bangkok, Thailand; 10 Department of Obstetrics and Gynecology, Faculty of Medicine, Ramathibodi Hospital, Mahidol University, Bangkok, Thailand; 11 Department of Pathology, Faculty of Medicine, Ramathibodi Hospital, Mahidol University, Bangkok, Thailand

**Keywords:** Acute chorioamnionitis, Amniotic fluid, Chorioamnionitis, Chorioamniotic membranes, *Enterococcus*, Hybrid genome assembly, Infection, Intraamniotic infection, Nanopore, Preterm labor

## Abstract

**Background:**

Preterm labor syndrome is associated with high perinatal morbidity and mortality, and intra-amniotic infection is a cause of preterm labor. The standard identification of causative microorganisms is based on the use of biochemical phenotypes, together with broth dilution-based antibiotic susceptibility from organisms grown in culture. However, such methods could not provide an accurate epidemiological aspect and a genetic basis of antimicrobial resistance leading to an inappropriate antibiotic administration. Hybrid genome assembly is a combination of short- and long-read sequencing, which provides better genomic resolution and completeness for genotypic identification and characterization. Herein, we performed a hybrid whole genome assembly sequencing of a pathogen associated with acute histologic chorioamnionitis in women presenting with PPROM.

**Results:**

We identified *Enterococcus faecium*, namely *E. faecium* strain RAOG174, with several antibiotic resistance genes, including vancomycin and aminoglycoside. Virulence-associated genes and potential bacteriophage were also identified in this genome.

**Conclusion:**

We report herein the first study demonstrating the use of hybrid genome assembly and genomic analysis to identify *E. faecium* ST17 as a pathogen associated with acute histologic chorioamnionitis. The analysis provided several antibiotic resistance-associated genes/mutations and mobile genetic elements. The occurrence of *E. faecium* ST17 raised the awareness of the colonization of clinically relevant *E. faecium* and the carrying of antibiotic resistance. This finding has brought the advantages of genomic approach in the identification of the bacterial species and antibiotic resistance gene for *E. faecium* for appropriate antibiotic use to improve maternal and neonatal care.

**Supplementary Information:**

The online version contains supplementary material available at 10.1186/s12864-023-09511-1.

## Background

Preterm labor is the leading cause of perinatal morbidity and mortality worldwide [1–8]. Two-thirds of preterm deliveries occur after the spontaneous onset of preterm labor, with either intact or ruptured membranes [7, 9–11]. Intraamniotic infection is causally linked to spontaneous preterm delivery/PPROM [12–16]. One of every three preterm infants is born to a mother with intraamniotic infection that is largely subclinical [12–16]. Microorganisms isolated from the amniotic fluid are similar to those found in the lower genital tract; therefore, an ascending pathway is considered the most frequent route of infection [17]. The most common microorganisms identified in the amniotic cavity in women presenting with preterm labor/PPROM include *Ureaplasma urealyticum*, *Mycoplasma hominis*, *Bacteroides* spp., *Gardnerella vaginalis*, *Neisseria gonorrhoeae*, *Chlamydia trachomatis*, *Trichomonas vaginalis*, and group B hemolytic streptococci [18]. In 30% of cases with intraamniotic infection, bacteria are identified in fetal circulation [19], resulting in FIRS [20, 21]. Such fetuses have multi-organ involvement and are at risk for long-term complications, such as cerebral palsy and chronic lung disease, underscoring that complications of infants born preterm are not only due to immaturity but also to the inflammatory process responsible for preterm labor [14, 21, 22]. Therefore, accurate identification of a causative pathogen is essential for the eradication of microbial invasion of the amniotic cavity with antibiotics [23–32].

In clinical medicine, identification of the presence of bacteria and bacterial species is based on cultivation and the use of biochemical phenotypes, together with broth dilution-based antibiotic susceptibility. However, such methods are time-consuming, and they could not provide the epidemiological aspect and genetic basis of antimicrobial resistance [33, 34]. Knowledge of the presence of specific bacterial species and AMR genes can guide decision-making to deliver or to treat intraamniotic infection with a particular antibiotic. In addition, the understanding of the specific microorganism and the AMR gene profile could be helpful to the neonatologist to tailor antimicrobial agents appropriate for each newborn [35, 36].

Our group recently reported the use of the 16S nanopore sequencing method, a long-read sequencing, for rapid identification of intraamniotic infection in patients with PPROM [37]. This method allows identification of bacteria at the species level within 5–9 h from DNA extraction, demonstrating that this sequencing technique is effective for clinical use in a timely manner. We have extended the study by performing whole genome sequencing since whole genome sequencing and comparative genomic analysis allow insightful information, i.e., microbiological diagnosis and infectious outbreak investigations [38–40]. Several studies utilize whole genome sequencing to identify causative pathogens from clinical specimens [39, 41]. Hybrid genome assembly, which is a combination of short- and long-read sequencing, provides better genomic resolution and completeness for genotypic identification and characterization [42]. A recent study demonstrated that hybrid assembly using Illumina and Nanopore sequencing elucidated genomic insight of multidrug-resistant bacteria and provided epidemiological data of the pathogen [43]. Herein, we performed hybrid whole genome assembly sequencing of a pathogen associated with acute histologic chorioamnionitis in women presenting with PPROM.

## Results

During the clinical microbiological laboratory investigation, the sample of the chorioamniotic membranes was cultured under an aerobic condition. When the culture was negative on day-3 after cultivation, the placental tissue was then cultured under an anaerobic condition. The bacteria were finally recovered and identified as *E. faecium* by conventional phenotypic methods on the 10^th^ day after anaerobic cultivation (13th day after specimen collection). However, antibiotic susceptibility test was not performed because the susceptibility test of anaerobic culture was done by request only according to our hospital protocol. The colonies were also collected for genomic DNA extraction. This isolate was listed as the *E. faecium* RAOG174 strain.

Whole genome sequencing was performed on colonies recovered from the anaerobic culture of chorioamniotic membranes. ONT sequencing and assembly delivered a total number of 3,150,009 bp, comprised of 11 contigs, largest contigs of 2,852,659 bp, N50 of 2,852,659 bp, and GC content of 37.8%. Genome annotation of *E. faecium* RAOG174, using Dfast software [44], resulted in 3,038 CDSs, 18 rRNAs, and 67 tRNAs. Species identification, using a whole genome sequence, agreed with *E. faecium* with ANI of 98.98%, calculated by fasANI. Additional whole genome analysis was performed to illustrate the molecular identification (Supplementary Figures. 1 and 2). The isolate was assigned to ST17, based on MLST scheme. Global genetic epidemiological analysis of *E. faecium* using bacWGSTdb 2.0 provided MLST-based typing similar to a previous analysis (ST17),however, the most closely related isolate was not available.

### Genome features

#### Resistance

Potential antibiotic resistance genes were predicted with CARD and ResFinder 4.1. Genes included several resistance mechanisms to beta-lactam, quinolone, aminoglycoside, macrolide, tetracycline, and vancomycin (Table 1). Mutations in *pbp5*, *parC* and *gyrA* genes were considered intrinsic resistance. The others were considered acquired resistance (Table 1 and Supplementary Table 1).

**Table 1:** The presence of genetic basis of antimicrobial resistance-associated genes in the *E. faecium* AOG174 genome

Antibiotic class	Genetic mechanism	Resistance mechanism	Genetic localization
Beta-lactam	*Pbp5* mutation	Target modification	Intrinsic
Aminoglycoside	*aac(6’)*	Drug modification	Acquired
Fluoroquinolone	*parC* and *gyrA* mutation *efm*	Target modification Efflux pump	Intrinsic Acquired
Tetracycline	*Tet (O)* gene	Target protection	Acquired
Glycopeptide	*vanHAX* gene	Target modification	Acquired
Macrolide	*erm* gene *efm* gene *msr(C)* gene	Target modification Efflux pump Efflux pump	Acquired Acquired Acquired

#### Detection of mobile genetic elements and phages

Using Mobile Element Finder, a total number of 61 IS elements were predicted (shown as the black arc in Supplementary Figure 2). The most abundant type of IS was identified as IS*Efa*11, followed by IS*Efa*5 and IS*Enfa*3. PHASTER identified 7 phage regions on the chromosome: 2 intact regions (PHAGE_Lister_2389 and PHAGE_Lister_LP_101) (red arcs in Supplementary Figure 1), 4 incomplete regions (PHAGE_Paenib_Xenia, PHAGE_Lactoc_bIL311, 2 regions of PHAGE_Bacill_vB_BtS_BMBtp14), and 1 questionable region (PHAGE_Entero_EFAP_1). CRISPRCasFinder identified 1 region that was similar to the CRISPR-Cas region, but it was categorized into “level 1,” which is unlikely.

#### Virulence

For the virulence-associated genes, the VFanalyzer and Virulence Finder identified a total of 22 virulence genes (Table 2). Of them, 12 genes were previously described in *E. faecium* DO. The other 10 genes were similar to previously defined virulence genes in other genera, including *Streptococcus*, *Staphylococcus*, *Listeria*, and *Vibrio*.

**Table 2:** List of predicted virulence-associated genes in *E. faecium* RAOG174, compared to the *E. faecium* DO genome

VF class	Virulence factors	Related genes	*E. faecium* RAOG174 (Prediction)	*E. faecium* strain DO
Adherence	Acm	*acm*	+	HMPREF0351_12281
	Ebp pili	*ebpA*	+	HMPREF0351_11394
		*ebpB*	+	HMPREF0351_11393
		*ebpC*	+	HMPREF0351_11392
		*srtC*	+	HMPREF0351_11391
	EcbA	*ecbA*	+	HMPREF0351_11828
	EfaA	*efaA*	+	HMPREF0351_10500
	Esp	*esp*	+	-
	Scm	*scm*	+	HMPREF0351_12673
	SgrA	*sgrA*	+	HMPREF0351_11523
	Streptococcal lipoprotein rotamase A (*Streptococcus*)	*slrA*	+	-
Anti-phagocytosis	Capsule	*cpsA/uppS*	+	HMPREF0351_11682
		*cpsB/cdsA*	+	HMPREF0351_11681
Biofilm formation	BopD	*bopD*	+	HMPREF0351_10415
Enzyme	Hyaluronidase	Undetermined	+	-
	Serine-threonine phosphatase (*Listeria*)	*stp*	+	-
Immune evasion	Capsule (*Staphylococcus*)	*capL*	+	-
	Capsule (*Streptococcus*)		+	-
		*rgpG*	+	-
Iron uptake	Periplasmic binding protein-dependent ABC transport systems (*Vibrio*)	*vctC*	+	-
Protease	Serine protease (*Streptococcus*)	*htrA/degP*	+	-
Surface protein anchoring	Lipoprotein diacylglyceryl transferase (*Listeria*)	*lgt*	+	-

## Discussion

This study demonstrates the first use of hybrid genome assembly to identify the potential virulence, the AMR gene profile, and the sequence type of bacterial pathogen associated with acute histologic chorioamnionitis in women presenting with PPROM. Identification of a causative pathogen is the key for obstetricians to administer proper antibiotics to the mother in order to eradicate intraamniotic infection or to prevent postpartum infectious complications such as endometritis and pelvic abscess [45]. Currently, cultivation-based species identification generally takes 48 h (including biochemical assay and antibiotic susceptibility test). Whole genome sequencing using nanopore method could reveal the result by, at least, 2 h with more informative data, including sequence type, antibiotic resistance-associated genes and virulence genes. In addition, an understanding of the accurate bacterial species and its genomic information is essential for neonatologists to initiate appropriate antibiotic agents to the neonate. This work demonstrates that the potential causative pathogen carried several genes associated with antibiotic resistance and that proper antibiotic selection was crucial in an individual case.

In this study, the patient presented with PPROM and suspected intraamniotic infection. The definite diagnosis of intraamniotic infection was difficult due to anhydramnios; therefore, the microbiologic work-up from amniotic fluid was unsuccessful. However, there was evidence of maternal systemic inflammation as shown by the elevated WBC and CRP as well as acute histologic chorioamnionitis and acute funisitis. The latter two represent the placental histologic landmarks of intraamniotic infection and FIRS [46]. Microbiological identification eventually recovered *E. faecium* from the anaerobic culture of chorioamniotic membranes.

*Enterococcus* species are gram-positive bacteria that are abundant in the gastrointestinal tract of a wide range of animals [47]. In humans, *Enterococci* are one of the earliest colonizers in the gut. Currently, more than 50 species of *Enterococcus* have been identified. However, a few species are clinically important, including *E. faecium* [48]. In general, *E. faecium* is a commensal and does not harm a healthy host, but it becomes pathogenic when the host is immunocompromised [49]. This bacterium is associated with urinary tract and surgical site infections and bacteremia, especially in a hospital-acquired setting [50]. In pregnancy, *Enterococcal* infection is uncommon; however, it has been identified in the amniotic fluid of women diagnosed with clinical chorioamnionitis [51]. Ncib et al. demonstrated that the presence of vaginal-derived *Enterococcus* spp. is associated with recurrent pregnancy loss [52], PPROM [53], and bacterial vaginosis in pregnant women [54]. Seliga-Siwecka and Kornacka reported that the presence of *E. faecalis* in amniotic fluid significantly increases the risk for acute placental inflammation, necrotizing enterocolitis, and bronchopulmonary dysplasia in neonates [55, 56].

To investigate genomic insight of the bacteria, hybrid assembly, using ONT and Illumina sequencing, was then performed to obtain the complete genome, which was matched to *E. faecium* with a high similarity index (ANI = 98.98%). Genome assembly resulted in a genome size of 2.8 Gbp and the GC content of 37.8%, similar to the previously reported genome characteristic of *E. faecium* (2.5–2.9 Gbp for genome size and 37.8–38.5% for GC content) [57]. Whole genome sequencing has been used as a diagnostic tool for clinical microbiology to understand genomic insight of the pathogen and has become a “gold standard” technique [58, 59]. In microbial genomics, hybrid genome assembly utilizes short- and long-reads to construct the complete or nearly complete genome with better resolution and less error [60–62]. Hybrid assembly uses long reads as scaffolds for short read-based contigs to be rearranged into a correct direction [63]. Khezri et al*.* demonstrated that hybrid genome assembly has better performance in constructing the complete genome, more accurately resulting in the identification of genes associated with virulence and drug resistance [42]. By incorporating the sequence information from both generations of sequencing, hybrid assembly can provide the sequence with the following improvements: reducing error rates, minimizing gaps, and unveiling sequences that are not covered by short-read sequencing alone [64].

It has been demonstrated that hybrid assembly could provide genomic importance for epidemiological study, particularly antibiotic resistance genes [65, 66]. The in silico sequence typing, using pubMLST, revealed that the *E. faecium* strain RAOG174 belongs to ST17. *E. faecium* ST17 is clinically relevant [67, 68] and is defined as an ancestor of CC17 [69]. The CC17 belongs to clade A, which is associated with human infection (as opposed to clade B, which is the commensal clade) [70, 71], however, it is later identified in non-clinical specimens, including wastewater and domestic animals [69]. The characteristics of CC17 are ampicillin and quinolone resistance, and most isolates have a putative pathogenicity island harboring the *esp* gene, which was also present in the isolate RAOG174 (Table 2) [72]. Some isolates additionally carry the *vanA* gene conferring vancomycin resistance [71]. The *vanA* typically situates in *vanHAX* gene clusters where *vanH, vanA*, and *vanX* encode for D-alanyl-D-lactate ligase, α-keto acid reductase, and Zn2 + -dependent d-Ala-d-Ala dipeptidase, respectively [73]. The isolate RAOG174 is likely ampicillin- and vancomycin-resistant due to the presence of *pbp* mutation and *vanHAX*, respectively (Table 1), although *vanA*-positive vancomycin-susceptible *E. faecium* was also previously identified [74, 75].

Our work revealed that IS and phages were predicted in the *E. faecium* RAOG174 genome. *E. faecium* is highly evolved and adaptable, as previously illustrated by its open pan-genome [76]. With this genomic characteristic, *E. faecium* can receive and donate genetic elements, including antibiotic resistance genes, with other cells or environments. In this study, major antibiotic groups, including beta-lactam, glycopeptide, aminoglycoside, fluoroquinolone, and macrolide, were predicted. AMR gene analysis identified point mutations and genes that are associated with antibiotic resistance, and these genes/mutations indicated intrinsic resistance and acquired resistance. *E. faecium* has been known to exhibit several antibiotic resistance phenotypes.

“Empirical antibiotics with ampicillin and erythromycin was administered for prolong latency period in women with preterm PROM. Postpartum period was uneventful. Neonate was diagnosed with suspected sepsis based on mother’s history (preterm PROM) and was administered with routine antibiotics (ampicillin and gentamicin) for 7 days.” The standard management of clinical chorioamnionitis is the administration of antibiotics and augmentation of labor [45, 77–79]. The American College of Obstetricians and Gynecologists recommended the use of ampicillin and gentamicin (ampicillin 2 g IV every 6 h combined with gentamicin 5 mg/kg every 24 h) whenever an intraamniotic infection is suspected or confirmed [80]. Such antibiotics would not have been effective against the *E. faecium* reported herein. For the neonates, antibiotic prescription is recommended only in symptomatic neonates and in neonates with risk of early-onset sepsis due to an avoidance of inappropriate antibiotic exposure [81, 82] with the standard regimen of ampicillin and gentamicin [83]. Interestingly, a case of early-onset sepsis caused by vancomycin-resistant *E. faecium* in a newborn, who was born from a mother without any sign of clinical chorioamnionitis, has been reported. The newborn was initially diagnosed with meconium aspiration syndrome and neonatal sepsis and then was treated with the standard antibiotic regimen for early-onset neonatal sepsis. Then antibiotic-resistant *E. faecium* was identified from the blood culture of the baby. Subsequently, the antibiotic was changed to linezolid according to its antibiotic susceptibility. The neonate was discharged in normal condition after receiving linezolid for 2 weeks [84].

The *vanA*-positive *E. faecium* is mostly associated with hospital-acquired infection. The carriage rates in a given community vary from 0%- to -13% depending on the geographic region [85–90], and community-acquired infections are rare. In our study, we could not identify the source of *E. faecium*. Our hospital has a standard protocol for regular surveillance of VRE. We confirmed that VRE was not detected in the clinical microbiology lab and the ward (where the patient stayed) during the duration of sample collection and patient’s hospitalization.

In general, VRE should not be identified in the labour room, according to the hospital’s infection control surveillance. According to the history that documented the patient had been previously admitted at her provider hospital prior to transferring to our hospital, the patient could have contracted VRE colonization from the previous hospital. As *E. faecium* is one of the colonizers in human gastrointestinal tract, the bacteria could migrate from maternal gut to maternal blood stream and then enter the chorioamniotic membranes transplacentally [91–93].

Alternatively, samples with low bacterial biomass, such as the amniotic fluid and placenta, are commonly vulnerable to bacterial contamination from the environment [94]. VRE is one of the most common laboratory contaminants, and the prevalence of VRE contamination ranges from 10%- to -60% [95, 96]. The most common contaminated sites are the work surface and the hands of the healthcare worker [97]. However, in our case, we believed that this is a true pathogen, as the patient had clear evidence of systemic maternal inflammatory response and her placenta showed acute chorioamnionitis as well as acute funisitis. In addition, she had a history of multiple hospital admissions prior to the last visit. Therefore, despite the possibility of lab contamination, the VRE strain RAOG174 could be the true pathogen associated with acute chorioamnionitis.

Our results suggest that the precise identification of a pathogen and its antibiotic susceptibility is essential for administration of appropriate antibiotics since the routine antibiotic regimen for chorioamnionitis is not effective against this microorganism. Whole genome sequencing, together with an established bioinformatic analysis pipeline, could provide more robust bacterial identification of causative pathogens. Importantly, one study illustrated that the whole genome sequencing approach took approximately 36 to 42 h, compared to conventional (cultivation-based) identification in 48 to 72 h [39]. It is important to note that the nanopore sequencing achieved nearly 30 × coverage of the VRE genomes within a sequencing run of only 40 min. With this depth of coverage, we were able to obtain similar contigs as observed in the hybrid assembly. Furthermore, the advantage of the long-read assembly lies in its rapidity. Our results show that the long-read assembly method can deliver results swiftly, suggesting its potential for future applications in clinical testing. These advantages may lead to appropriate antibiotic use that may enhance maternal and neonatal care by reducing identification time and by providing more precise microorganism determination.

## Conclusions

Chorioamnionitis is a global health care problem, and the accurate identification of the causative pathogen is beneficial for the pregnant woman and her neonate. This study is the first to demonstrate the use of hybrid genome assembly and genomic analysis to identify *E. faecium* ST17 as a pathogen associated with acute histologic chorioamnionitis. The analysis provided several antibiotic resistance-associated genes/mutations and mobile genetic elements. Although the source of infection could not be identified, the occurrence of the vancomycin-resistant gene carrying *E. faecium* ST17 raised awareness of the colonization and the infection of highly resistant bacteria in a pregnancy-related setting in which resistance identification is critical. This finding spotlights the advantages of the hybrid genome assembly approach in bacterial species and in antibiotic-resistance gene identification for appropriate antibiotic use to improve maternal and neonatal care.

## Methods

### Patient history and clinical information

A 39-year-old para 0 at 30^+5^ weeks of gestation, presented to the Labor and Delivery Unit at our hospital due to leakage of fluid and the onset of abdominal cramps every 5 min. At 28^+5^ weeks of gestation, she had an episode of vaginal bleeding and was diagnosed with placenta previa. The patient was admitted for 3 days at her provider hospital and vaginal bleeding was lessened. At 30 weeks of gestation, she was re-admitted at her provider hospital due to a second episode of vaginal bleeding. Five days later, she experienced rupture of membranes and was transferred to our hospital. Upon arrival, her vital signs were normal without a fever or tachycardia. Transabdominal ultrasound demonstrated a single viable fetus with an estimated fetal weight of 1,507 g (percentile 50–90) and anhydramnios. The patient’s laboratory examination showed anemia (Hb 8.7 g/dL) and an elevated white blood cell (WBC) count as well as C-reactive protein (CRP) [WBC 29,990 cells/microliters (neutrophils 91%) and CRP 138.35 mg/L]. The diagnosis was PPROM, placenta low-lying with preterm labor. Expectant management was undertaken with the administration of steroids to promote fetal lung maturity, antibiotic agents (ampicillin and erythromycin) to prolong the latency period, and magnesium sulfate for tocolysis. Five days after admission, the patient developed regular uterine contractions every 5 min. A Cesarean section was performed due to preterm labor with placenta previa. A female fetus was delivered. The neonate was diagnosed with suspected sepsis (according to mother history) and was administered ampicillin and gentamicin for 7 days. Placental histopathology demonstrated acute histologic chorioamnionitis with acute funisitis. The placental chorioamniotic membranes culture demonstrated *Enterococcus faecium* (*E. faecium*). This enrollment and the use of clinical specimens of this patient were approved by the Institutional Research Board of the Faculty of Medicine, Ramathibodi Hospital, Mahidol University (COA.MURA2021/254 and COA.MURA 2022/675)*.*

### Placental cultivation

Placental tissue derived from the chorioamniotic membranes was obtained after cesarean delivery with an aseptic technique and kept in a sterile, capped container within the sterile operating field. The placental sample was then brought directly to a biosafety cabinet located at the microbiology unit in the same building by using a standard transportation process. The placental tissue was divided into approximately 1 cm^2^ in size and inoculated on blood agar, MacConkey agar, and chocolate agar under an aerobic condition and on thioglycolate broth under an anaerobic condition.

### Isolation and sequencing

Bacteria were recovered from the placental tissue by cultivating under aerobic and anaerobic conditions, using an aseptic technique throughout the processes. Genomic DNA was extracted from bacterial colonies obtained from the anaerobic culture. The purity of the extracted DNA was observed by a Nanodrop Spectrophotometer (Thermo Fisher Scientific, USA), and the quantity was checked by a Qubit® 4.0 Fluorometer (Invitrogen, USA). Whole genome sequencing was conducted with single molecular sequencing (ONT UK) and Illumina short-read sequencing. Briefly, DNA library preparation was performed by using the Rapid Barcoding Sequencing Kit (SQK-RBK004; ONT). A total of 200 ng of genomic DNA was cleaved with transposase enzyme to produce chemically modified ends and a barcode was added to each DNA sample, finally ligated with an adapter. The library was loaded into the R9.4.1 flow cell (FLO-MIN106 version; ONT) and sequenced with the GridION device (ONT) with 72-h sequencing. For Illumina short-read sequencing, 150-bp paired-end libraries were prepared with a TruSeq DNA PCR-free Kit and sequenced with the Illumina™ Novaseq sequencer (Illumina Inc., San Diego, CA, USA). To obtain high-quality reads, adapter sequences were trimmed by using Skewer v0.2.2. The sequence data from both platforms were combined for hybrid genome assembly. This protocol was approved by Institutional Biosafety Committee of the Faculty of Medicine at Ramathibodi Hospital, Mahidol University (RAMA-IBC 2022–009).

### Assembly and annotation

A hybrid genome assembly approach was selected to produce a de novo assembly of the *E. faecium* genome. This approach involved the use of Illumina short-read and Nanopore long-read sequencing technologies to produce a complete and accurate genome assembly. The hybrid genome assembly procedures were depicted as a series of flowing steps. For short reads QC, we used the Fastp v0.20.1 tool to trim sequencing adapters and low-quality reads, followed by quality assessment with FastQC v0.11.8 (http://www.bioinformatics.babraham.ac.uk/projects/fastqc/). For long reads, raw signals were processed, demultiplexed, and adapter trimmed, using Guppy v6.2.1 with the super accurate model (–c dna_r9.4.1_450bps_sup.cfg -r –trim_barcodes –barcode_kits SQK-RBK004) and Porechop v0.2.4 (https://github.com/rrwick/Porechop). The quality of the ONT raw reads was assessed by using NanoPlot v1.28.1. The reads were filtered by NanoFilt v2.5.0 [98], based on a mean quality score of 9, and only reads with a length of 1,000 bases were retained for the de novo assembly. Finally, we constructed the genomes with Unicycler v0.4.8 [63], which incorporates hybrid assembly, correction, circularization, and rotation to produce high-quality genome assemblies. Gene annotation was performed in Dfast [44]. MLST assignment was performed by using FastMLST software [99]. Analysis of mobile genetic element, phage, potential antimicrobial resistance genes, and virulence-associated genes was executed by using the Mobile Element Finder, PHASTER, ResFinder version 4.1, CARD and MLST service, respectively [100–103]. CRISPRCasFinder was used to determine potential CRISPR-Cas region on the chromosome [104]. Virulence-associated genes were obtained by using VFanalyzer [105] and Virulence Finder [106]. As E. faecium is clinically important, especially in hospital-associated infection, genomic epidemiology analysis was performed via web-based platforms in bacWGSTdb 2.0 [107]. The visualization of comparative genomics analysis was completed in BRIG [108]. Phylogenetic tree was constructed by using PhyML with 1000 bootstrap repeats [109] and visualized with Figtree version 1.4.4 (http://tree.bio.ed.ac.uk/software/figtree/). The tree was constructed using type strains: *Enterococcus thailandicus* DSM 21767 (Accession no:SAMN03267187), *Enterococcus ratti* DSM 15687 (Accession no:SAMN03267184), *Enterococcus mundtii* DSM 4838 (Accession no:SAMN03267178), *Enterococcus xinjiangensis* JCM 30200 (Accession no:SAMD00255146), *Enterococcus lactis* DSM 23655 (Accession no:IMG ID 2928549275), *Enterococcus lactis* CCM 8412 (Accession no:SAMD00255145), *Enterococcus villorum* NBRC 100699 (Accession no:SAMD00166261), *Enterococcus porcinus* ATCC 700913 (Accession no:SAMN02596958), *Enterococcus hirae* ATCC 9790 (Accession no:SAMN02604142), *Enterococcus canis* NBRC 100695 (Accession no:SAMD00046312), *Enterococcus casseliflavus* NBRC 100478 (Accession no:SAMD00045727), *Enterococcus durans* NBRC 100479 (Accession no:SAMD00045728), *Enterococcus faecium* NBRC 100486 (Accession no:SAMD00045730), *Enterococcus gallinarum* NBRC 100675 (Accession no:SAMD00045734).

## Supplementary Information

**Additional file 1: Supplementary Table 1.** The list of point mutation of *pbp5* gene, *gyrA* gene and *parC* gene identified in the genome of Entercoccus faecoum strain RAOG174. **Supplementary Figure 1.** A maximum likelihood phylogenetic tree with 1,000 bootstrap repeats constructed by using a whole genome sequence of the *Enterococcus faecium* strain RAOG174 (as highlighted in red). Only bootstrap support >75% was shown on a tree.** Supplementary Figure 2.** Comparative genomic visualization of the* Enterococcus faecium* (*E. faecium*) strain RAOG174 constructed with the Blast Ring Image Generator. Pink ring represents the *E. faecium* strain SRR24. Green ring indicates the *E. faecium* strain NBRC100486. Dark blue ring shows the *E. faecium* strain RAOG174. Black boxes represent the Insert Sequence (IS). Red boxes represent the intact phage regions.

## Data Availability

The genome used in this study was available in the Genbank repository (BioProject PRJNA930917; Reviewer’s link is https://dataview.ncbi.nlm.nih.gov/object/PRJNA930917?reviewer=rn64h3763n6525qpdvobsb0igc).

## Abbreviations

AMR: Antimicrobial resistance
ANI: Average Nucleotide Identity
BRIG: BLAST Ring Image Generator
CARD: Comprehensive Antibiotics Resistance Database
CC: Clonal complex
CRISPR: Clustered Regularly Interspaced Short Palindromic Repeats
CRP: C-reactive protein
FIRS: Fetal systemic inflammatory response syndrome
MLST: Multi-locus sequence typing
ONT: Oxford Nanopore Technologies
PPROM: Preterm prelabor rupture of membranes
ST: Sequence Type
VRE: Vancomycin-resistant *E. faecium*
WBC: White Blood Cell
