# Supplementary material for: Genomic analysis of Enterococcus faecium strain RAOG174 associated with acute chorioamnionitis carried antibiotic resistance gene: is it time for precise microbiological identification for appropriate antibiotic use?
Source: BMC Genomics. 2023 Jul 19;24:405. doi: 10.1186/s12864-023-09511-1 (PMC10354890; doi:10.1186/s12864-023-09511-1)

**Supplementary data**

**Supplementary Table 1**: The list of point mutation of *pbp5* gene, *gyrA* gene and *parC* gene identified in the genome of Entercoccus faecoum strain RAOG174

| Gene | Mutation | Resistance | PMID |
| --- | --- | --- | --- |
| pbp5 | p.V24A | Ampicillin | 25182648 |
|  | p.S27G | Ampicillin | 25182648 |
|  | p.R34Q | Ampicillin | 25182648 |
|  | p.G66E | Ampicillin | 25182648 |
|  | p.A68T | Ampicillin | 25182648 |
|  | p.E85D | Ampicillin | 25182648 |
|  | p.E100Q | Ampicillin | 25182648 |
|  | p.K144Q | Ampicillin | 25182648 |
|  | p.T172A | Ampicillin | 25182648 |
|  | p.L177I | Ampicillin | 25182648 |
|  | p.D204G | Ampicillin | 25182648 |
|  | p.A216S | Ampicillin | 25182648 |
|  | p.T324A | Ampicillin | 25182648 |
|  | p.M485A | Ampicillin | 25182648 |
|  | p.N496K | Ampicillin | 25182648 |
|  | p.A499T | Ampicillin | 25182648 |
|  | p.E525D | Ampicillin | 25182648 |
|  | p.E629V | Ampicillin | 25182648 |
|  | p.P667S | Ampicillin | 25182648 |
| gyrA | p.S83I | Nalidixic acid, Ciprofloxacin | 9527801 |
| parC | p.S80I | Nalidixic acid, Ciprofloxacin | 12373497 |

**Supplementary Figure 1**. A maximum likelihood phylogenetic tree with 1,000 bootstrap repeats constructed by using a whole genome sequence of the *Enterococcus faecium* strain RAOG174 (as highlighted in red). Only bootstrap support >75% was shown on a tree.

**Supplementary Figure 2.** Comparative genomic visualization of the *Enterococcus faecium* (*E. faecium*) strain RAOG174 constructed with the Blast Ring Image Generator. Pink ring represents the *E. faecium* strain SRR24. Green ring indicates the *E. faecium* strain NBRC100486. Dark blue ring shows the *E. faecium* strain RAOG174. Black boxes represent the Insert Sequence (IS). Red boxes represent the intact phage regions.


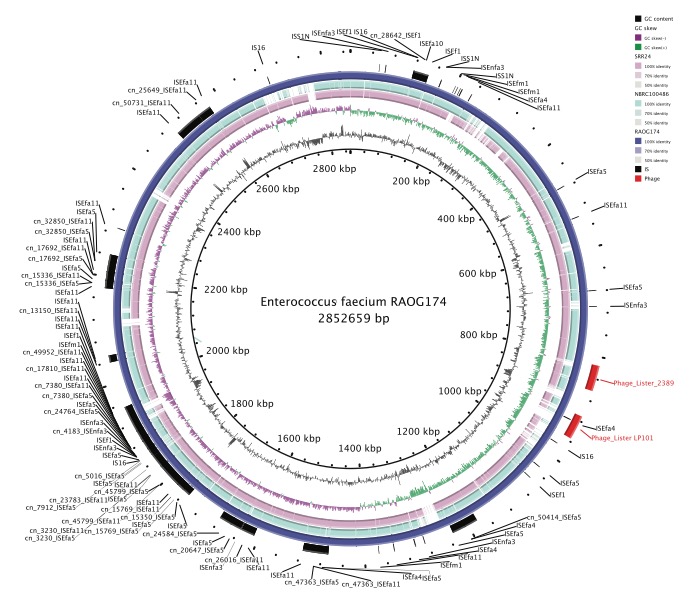

Supplement: Supplementary file 1 — Additional file 1: Supplementary Table 1. The list of point mutation of pbp5 gene, gyrA gene and parC gene identified in the genome of Entercoccus faecoum strain RAOG174. Supplementary Figure 1. A maximum likelihood phylogenetic tree with 1,000 bootstrap repeats constructed by using a whole genome sequence of the Enterococcus faecium strain RAOG174 (as highlighted in red). Only bootstrap support >75% was shown on a tree. Supplementary Figure 2. Comparative genomic visualization of the Enterococcus faecium (E. faecium) strain RAOG174 constructed with the Blast Ring Image Generator. Pink ring represents the E. faecium strain SRR24. Green ring indicates the E. faecium strain NBRC100486. Dark blue ring shows the E. faecium strain RAOG174. Black boxes represent the Insert Sequence (IS). Red boxes represent the intact phage regions. [file 12864_2023_9511_MOESM1_ESM.docx]
